# Automated detection of GFAP-labeled astrocytes in micrographs using YOLOv5

**DOI:** 10.1038/s41598-022-26698-7

**Published:** 2022-12-23

**Authors:** Yewen Huang, Anna Kruyer, Sarah Syed, Cihan Bilge Kayasandik, Manos Papadakis, Demetrio Labate

**Affiliations:** 1 Department of Mathematics, University of Houston, Houston, TX USA; 2 Department of Computer Engineering, Istanbul Medipol University, Istanbul, Turkey; 3 Division of Pharmaceutical Sciences, University of Cincinnati, Cincinnati, OH USA

**Keywords:** Neuroscience, Mathematics and computing

## Abstract

Astrocytes, a subtype of glial cells with a complex morphological structure, are active players in many aspects of the physiology of the central nervous system (CNS). However, due to their highly involved interaction with other cells in the CNS, made possible by their morphological complexity, the precise mechanisms regulating astrocyte function within the CNS are still poorly understood. This knowledge gap is also due to the current limitations of existing quantitative image analysis tools that are unable to detect and analyze images of astrocyte with sufficient accuracy and efficiency. To address this need, we introduce a new deep learning framework for the automated detection of GFAP-immunolabeled astrocytes in brightfield or fluorescent micrographs. A major novelty of our approach is the applications of YOLOv5, a sophisticated deep learning platform designed for object detection, that we customized to derive optimized classification models for the task of astrocyte detection. Extensive numerical experiments using multiple image datasets show that our method performs very competitively against both conventional and state-of-the-art methods, including the case of images where astrocytes are very dense. In the spirit of reproducible research, our numerical code and annotated data are released open source and freely available to the scientific community.

## Introduction

Astrocytes, a glial subtype named for their star-shaped morphology, have been the focus of increasing interest in the scientific literature due to a number of recent discoveries revealing a prominent role within the central nervous system (CNS)^1–3^. Astrocytes provide structural and functional support to neurons through control of blood flow^4^, regulation of neuronal activation through extracellular ion concentrations^5^, regulation of neuronal energy dynamics through the transfer of lactate^6^, and modulation of synaptic transmission via the release of gliotransmitters such as dopamine^7,8^, D-serine^9,10^, GABA^11^, glutamate^12^ and ATP^13,14^. Astrocytes are also active players in neuronal survival after brain injury, playing roles in neurogenesis, synaptogenesis and glial scar formation following traumatic brain injury^15^. The diversity of astroglial functions in the brain, and their critical roles in signaling with various cellular partners, including neurons, other glia, and cellular components of the vasculature, is reflected by their morphological complexity and unique shapeshifting properties. For instance, reactive astrogliosis—a process occurring in response to traumatic brain injury or neurological disease—is typically accompanied by molecular, cytoskeletal and functional changes that play a key role in disease outcome^16^. Astrocytes have a striking ability to swell and shrink in size in order to reduce neurotransmitter spillover and increase transmitter concentration within the synaptic cleft. For example, astrocytes undergo morphological plasticity in response to neurotransmitter release in order to shape synaptic activity and behavior^17–20^. The functional relevance of morphological changes in astrocytes is further supported by studies relating cell morphology to the expression of markers of astrocyte activation^21–23^.

Because morphological alterations in astrocytes are highly correlated with brain injury and various pathologies in the CNS, having access to quantitative methods that can efficiently detect and accurately quantify such morphological alterations is expected to play a fundamental role in filling existing knowledge gaps and advancing the development of new therapeutics. Unfortunately, progress in developing such quantitative methods has been slow due to the morphological complexity of astrocytes and their remarkable heterogeneity (i.e. their broad variation in size, shape and arborization, depending on location and physiological conditions). As a result, it is extremely difficult to adapt existing general-purpose algorithms for biomedical image analysis of astrocytes and other glial cells.

In this paper, we focus on the critical task of automated detection of astrocytes.

There are currently a relatively small number of image analysis methods targeted to astrocytes or other glial cells. The simplest methods adapt classical intensity thresholding techniques to identify individual astrocytes, often stained using GFAP as a marker. Healy et al.^24,25^ reviewed several automated thresholding strategies available as plugins of the widely-used FIJI software^26^ and found that the performance of threshold-based segmentation of fluorescent images of astrocytes and other glial cells “varies considerably in quality, specificity, accuracy and sensitivity with entropy-based thresholds scoring highest for fluorescent staining”. The main drawback of this approach is that performance is inconsistent and often unpredictable as it depends on a multiplicity of factors including conditions of acquisition, cell density and noise level, so that a laborious manual tuning of parameters is required to ensure satisfactory results. Additionally, thresholding methods are unable to process images where astrocytes are dense, failing to separate contiguous cells, and instead merging multiple cells together. To deal with images containing a dense population of astrocytes, Suwannatat et al.^27^ have proposed a probabilistic method that uses random walks to separate cells^28^. This method, however, uses a manual approach to detect each cell and its performance degrades rapidly if more than two cells are entangled. Another approach by Kulkarni et al.^29^ identifies astrocytes in fluorescent images using the nuclear marker DAPI, and then traces cell arbors using a tracing method called local priority based parallel (LPP) tracing. However, this method does not generate voxel-level segmentation and does not address the problem of cell separation. More recently, with the emergence of methods from machine learning in the biomedical field, a few authors have introduced learning-based algorithms for astrocyte detection. The first such method is the FindMyCell algorithm by Suleymanova et al.^30^, that applies the deep learning platform of DetectNet-Caffe and was shown to provide accurate astrocyte detection results on a specific dataset, outperforming more traditional methods. However, the DetectNet platform requires a large number of training samples to compute a satisfactory classification model (about 1000 training images were used in the original paper). Another drawback of this approach is that the DetectNet-Caffe platform, introduced in 2013, is less flexible than other deep learning libraries that have emerged more recently, such as PyTorch and Tensorflow, with the result that FindMyCell cannot be run using current software libraries. Another recent algorithm for astrocyte detection is GESU-net^31^, an algorithm that combines a geometric descriptor and a convolutional neural network for the task of detection and segmentation of astrocytes. This method was also reported to provide accurate detection, outperforming more traditional methods.

In this paper, we present an innovative algorithm for the automated detection of GFAP-labeled astrocytes in micrographs based on YOLO^32^—specifically its version YOLOv5—a powerful deep learning platform that can be customized to deal with a range of object detection tasks. A major advantage of this platform is its extreme flexibility which allows one to easily select the size of the deep learning architecture to match the complexity of the object detection task so that the network can be trained with a relatively small number of annotated images. Nonetheless, YOLOv5 offers a very sophisticated supervised learning framework that requires a laborious and computationally intensive procedure to compute the specific classification models needed to address the desired object detection task. As we will show below, the design of our astrocyte detection algorithm based on YOLOv5 requires not only an extensive training process but also the careful optimization of a large number of hyperparameters to achieve competitive performance.

To demonstrate the ability of our astrocyte detection approach, we tested its performance on a large set including brightfield and fluorescent micrographs of GFAP-labeled astrocytes. While the brightfield image set was already available in the literature, our fluorescent image set is released here for the first time to the scientific community. This set contains fluorescent images of astrocytes taken from different subregions of the nucleus accumbens and under different physiological conditions, resulting in images with a wide range of cell densities and astrocytes exhibiting a moderate level of heterogeneity, hence making the task of automated astrocyte detection more challenging.

Our numerical results show that our astrocyte detection algorithm based on YOLOv5 is extremely competitive against conventional and state-of-the-art methods in terms of accuracy and computational efficiency. By providing a highly accurate and automated framework for the detection of astrocytes, our method provides a critical tool for the quantitative analysis of astrocytes that can be directly applied to measure pattern of spatial distribution and will facilitate the extraction of morphological features that could be used to cluster cells with comparable phenotypic characteristics or identify or classify cells into subpopulations.

In the spirit of reproducible research, our Pytorch code is released open source together with our annotated fluorescent image dataset. Given the fact that very few image datasets of astrocytes are currently available in the literature, we believe that releasing this annotated dataset that can be used to benchmark algorithms for astrocyte detection will be particularly beneficial to the research community.

## Materials and Methods

### Image datasets

For the development and demonstration of our algorithms, we considered the following two image datasets.

#### BBBC dataset

This set consists of 1039 brightfield images of astrocytes from different subregions of adult rat brain containing chromogen-based staining against GFAP that were acquired using $20\times$ magnification. Each image has size $990 \times 708$ pixels and, in total, the images contain about 15,000 cells. This set is publicly available as the image set BBBC042^33^ from the Broad Bioimage Benchmark Collection (https://bbbc.broadinstitute.org/BBBC042). Annotations for the cell locations in these images are included and available at the website indicated above. However, as we observed that a number of astrocytes were not annotated in some images, we revised the annotation file.

#### Kruyer dataset

This image set was acquired in the laboratory of Dr. Kruyer from the Department of Neuroscience at the Medical University of South Carolina and we release it here for the first time. This set consists of 211 GFAP-stained fluorescent images of astrocyte cells from 5 different subregions of the nucleus accumbens of adult rats, namely, the anterior core, posterior core, dorsomedial shell, medial shell, and lateral shell. In addition, micrographs were collected from rats following various behavioral perturbations. For each brain region, images were generated from 3 groups of animals, one group treated with intravenous saline, one group having undergone extinction training following heroin self-administration, and one group undergoing cue-induced heroin relapse after withdrawal. Images were acquired using 20x magnification and contain about 4,200 cells in total whose locations were manually annotated by domain experts without prior knowledge of our detection results. To annotate the images we used VGG Image Annotation (VIA), an open source16 manual annotation software that allows the user to manually draw a rectangular box around an object in an image and save the corresponding coordinates^34,35^. Images in this dataset have different sizes: $987 \times 987$, $774 \times 774$, $1014 \times 1014$ or $1058 \times 1058$ pixels.

The Kruyer image set and corresponding annotations are released together with our code and documentation in: https://github.com/yewen/AstrocyteDetection.

The preparation of the Kruyer image set is described below.

### Preparation of Kruyer image sets

After undergoing various behavioral protocols, 15 male and female Long Evans rats were perfused transcardially with 4% paraformaldehyde and brains were incubated in 4% paraformaldehyde overnight at 4C. Tissue was sliced coronally at 50 μm using a vibrating blade microtome (Leica Microsystems) and tissue slices were stored in glycerol-based media at 4C. Slices containing the nucleus accumbens were incubated in 1X phosphate-buffered saline (PBS) with 2% Triton X-100 for 15 minutes at room temperature before incubation in PBS with 0.2% Triton X-100 (PBST) with 2% normal goat serum (block) for 1 hour at room temperature shaking gently. Slices were then incubated in primary antibody (chicken anti-GFAP, Abcam, ab4674) at 1:1000 in block for 24 h at $4\;^\circ$C shaking gently. Tissue was rinsed 3 times for 5 min each in PBST before overnight incubation in fluorescently conjugated secondary antibody (AlexaFluor) in PBST at room temperature. After rinsing tissue 3 times for 5 min each in PBST, tissue was mounted onto glass slides using ProLong Gold Antifade Reagent and imaged using a Leica Stellaris 5 confocal microscope using a 20x objective lens. 16-bit image stacks, with about 40 images per stack, were collected at 600 Hz with a frame size of $1024\times 1024$ and a 0.5 μm step size, for a final image size 579.44 μm^2^ and pixel size of 566.4 nm^2^. Imaging was conducted such that each image reflected only one predetermined nucleus accumbens subregion (either anterior core, posterior core, dorsomedial shell, medial shell, or lateral shell) by comparison to^36^. Acquired image stacks were finally converted into planar images by maximum projection before applying our astrocyte detection algorithm.

#### Ethics declaration for animal experiments

Animal procedures and experiments were in line with standards and regulations to reduce the suffering of the animals approved by the Institutional Animal Care and Use Committee (IACUC) of the Medical University of South Carolina (MUSC). MUSC complies with the USDA Animal Welfare Act (Public Law 89-544) as amended by PL91-579 (1970), PL94-279 (1976) and 45 CFR37618 (1980); Health Research Extension Act of 1985 (Public Law 99-158); follows the Public Health Service Policy on Humane Care and Use of Laboratory Animals (revised September, 1986); and the Guide for the Care and Use of Laboratory Animals (revised September 1986); the Guide for the Care and Use of Laboratory Animals DHEW (NIH) 85-23 revised 1985. All authors complied with the ARRIVE guidelines.

### Object detections using YOLOv5

In this paper, we propose a novel deep learning approach for the automated detection of astrocytes based on YOLOv5. YOLO (You Only Look Once) was designed^32^ to predict what objects are present in an image and where they are, and addresses object detection as a regression problem. With respect to other object detection algorithms, it offers a number of advantages:

1. Flexibility. YOLOv5 offers the ability to train the network using images of different dimensions and colors, which makes the training process more flexible.
2. Customization. Another feature of YOLOv5 is that it allows customization of network architecture and selection of the storage size model depending on the complexity of the desired model. During training, multiple options are available for data-augmentation and optimization.
3. Convenience. With respect to the earlier versions of the software, YOLOv5 is implemented in PyTorch, a widely used Python-based scientific computing package that has become one of the preferred deep learning research platforms due to its great flexibility and computational efficiency^37^. PyTorch is currently the most widely deep learning and artificial intelligence library in the research community (https://www.assemblyai.com/blog/pytorch-vs-tensorflow-in-2022/).

**Figure 1:**
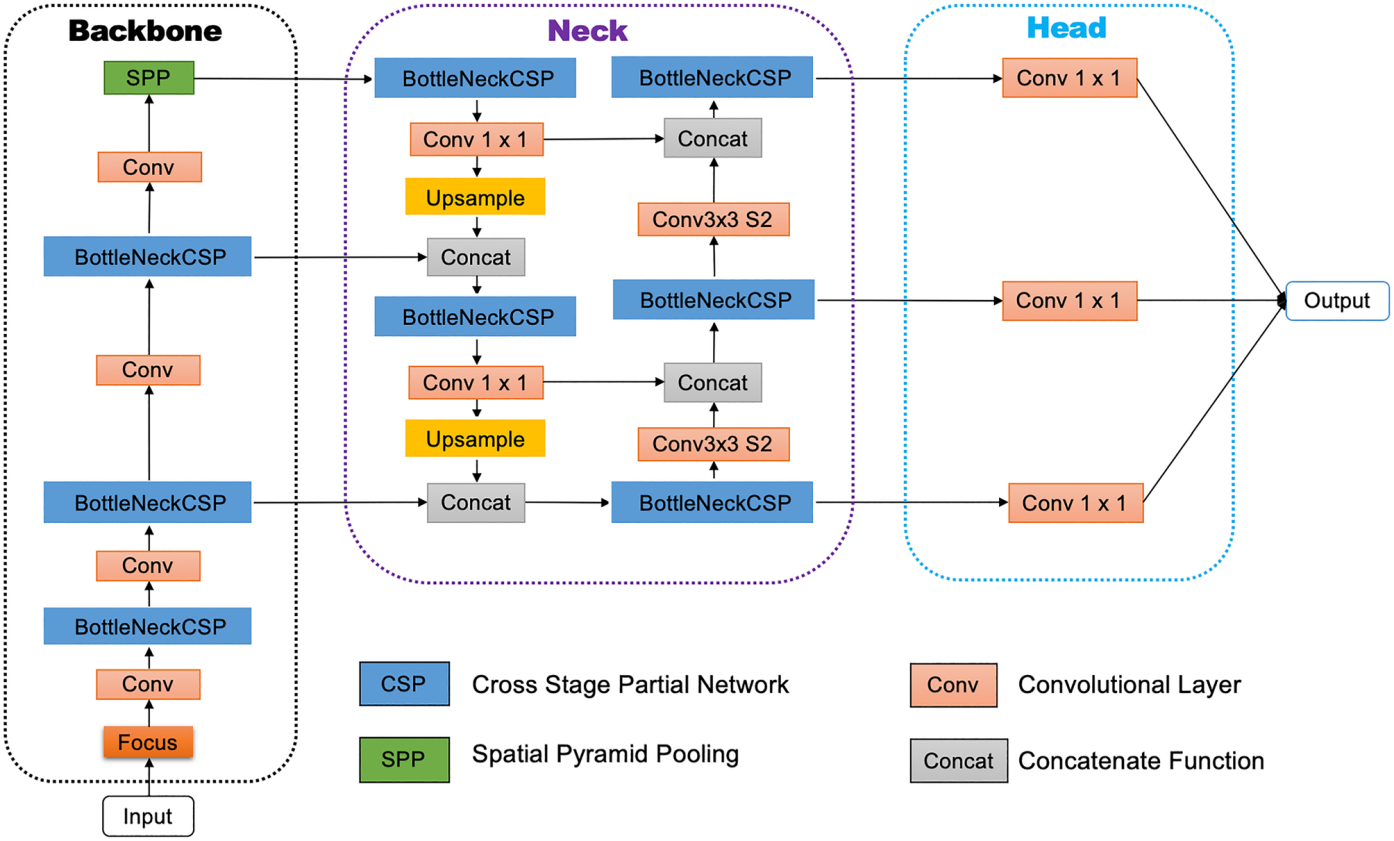
Network architecture. The figure shows the architectural diagram of YOLOv5 (default setting). It consists of three sections: (1) Backbone, (2) Neck, and (3) Head. Following preprocessing, images are first input to the Backbone for feature extraction, then are fed to the Neck for feature fusion and finally processed by the Head network to generate a model and output detection results (class, score, location, size).

The general network architecture of YOLOv5 is shown in Fig. 1 and is composed of three sections: Backbone, Neck and Head. We remark that, preceding the Backbone stage, an input terminal is devoted to image preprocessing, a stage including data augmentation^38^ and adaptive image padding. In addition, to deal with different image sizes, YOLOv5 implements an adaptive anchor frame calculation on the input image which allows the initial anchor frame size to be set automatically. The Backbone section is designed to extract feature maps of different sizes from the input image by multiple convolution and pooling that are implemented by incorporating cross stage partial network (CSPNet)^39^ into Darknet and creating CSPDarknet as its backbone. Next, the Neck section applies path aggregation network (PANet)^40^ to boost information flow. PANet adopts a feature pyramid network (FPN) structure with enhanced bottom-up path to improve the propagation of low-level features. Next, the Head section generates image features of 3 different sizes, namely $18 \times 18$, $36 \times 36$ and $72 \times 72$ pixels, so that the algorithm can capture structures occurring at different spatial scales in the image. The extraction of multiscale image features, which is a well-established approach in image analysis, is important to ensure accurate detection for complex objects such as astrocytes, as they include elongated processes occurring over a range of spatial scales (in our images, astrocyte processes exhibit lengths varying typically between 10 and 50 pixels).

Based on the predictions of the Head section, the output layer produces bounding box coordinates for the detected objects together with **detection scores** and **class probabilities**. In our case, since there is only one class of objects (the astrocytes) the output only consists of bounding box coordinates with the corresponding detection scores, where a detection score is a number between 0.0 and 1.0. The value of the detection score measures the level of confidence that the bounding box actually contains an object of interest, with detection score 0.0 being the the lowest level of confidence and 1.0 being the highest one. Finally, the user chooses a **confidence threshold** to decide which bounding boxes to accept.

As observed above, YOLOv5 allows the user to customize the architecture. YOLOv5 includes five architectures, named P5 models, which vary by their parameters size: YOLOv5n, YOLOv5s, YOLOv5m, YOLOv5l, YOLOv5x. Each of these models applies a different multiplier to the depth and width of the model, meaning that the overall structure of the network remains constant but the size and complexity of each model varies. While working on this project, a new set of architecture, called P6 models, was introduced which include a modified Backbone section and an extra output layer. These new architectures are designed to work more efficiently with images of larger size, e.g., $1280 \times 1280$ pixels.

For our detection tasks, which concerns a single object class and a relatively small image set, we adopted an intermediate architecture size, namely, the YOLOv5s architecture (from the P5 models) for the BBBC dataset and the YOLOv5s6 (from the P6 models) for the Kruyer dataset. We found experimentally that these intermediate-size architectures performed better than smaller or larger ones. We also found that the YOLOv5s6 performed better than the YOLOv5s architecture for the Kruyer dataset and explain this improved performance with the observation that the Kruyer dataset includes images of larger size as compared with the BBBC dataset.

We also remark that YOLOv5 network structure is regularly upgraded and updated. The so-called SPP structure^41^ that was used in this paper is currently being replaced by the SPPF structure^42^ which is claimed to have improved calculation efficiency.

While YOLOv5 can be applied to detect multiple object classes, for our purpose here we selected the setting corresponding to the detection of a single object class.

#### Loss function

As for any supervised learning platform, the loss function plays a critical role in determining the quantitative criteria by which to assess the success of the object detection task. The loss function of the YOLOv5 model includes three functional components^43^:

$$\begin{aligned} \text{ Loss }=L_{box}+L_{obj}+L_{cls} \end{aligned}$$

where $L_{box}$ is the *location loss*, $L_{obj}$ is the *objectness loss* and $L_{cls}$ is the *class loss*.

The location loss $L_{box}$, also known as the *bounding box loss*, controls the error associated with the location and size of the bounding box containing the object of interest (an astrocyte, in our case). The location loss measures the Intersection over Union (IoU) and is available in three forms: GIoU^44^, DIoU^45^ or CIoU^46^. We recall that IoU takes the set *A* of detected object pixels and the set *B* of true object pixels and calculates $IoU(A,B) = \frac{A \cap B}{A \cup B}$; the larger *IoU*(*A*, *B*), the better the detection. In our work, we adopted the CIoU location loss as we found heuristically that it provided the best detection performance. The objectness loss $L_{obj}$ controls the error of detecting the object of interest and is calculated using the Binary Cross Entropy (BCE) with logits loss. Finally, the class loss $L_{cls}$ controls the error associated with class assignment, in case there are multiple classes of objects to be detected and is realized using the BCE loss; since in our detection task there was only one class of interest (i.e., astrocytes), this component of the loss function was set to zero.

Convergence of the loss function in YOLOv5 is associated with the maximization of a *Fitness Function* that is defined as a weighted combination of four loss function metrics: (precision, recall, mAP@0.5, mAP@0.5:0.95). Here mAP denotes the mean Average Precision over different IoU thresholds^47^. The default Fitness Function assigns weights (0, 0, 0.10, 0.90), meaning that mAP@0.5 contributes 10% of the weight and mAP@0.5:0.95 contributes the remaining 90%. However, after extensive numerical experiments, we found that, for our data, we achieved improved convergence of the loss function during training by assigning the weights of the Fitness Function as (0.5, 0.5, 0, 0).

### Hyperparameter setting and network training

As for any learning based approach, training and validation are needed to learn the network parameters needed to compute an astrocyte detection model based on YOLOv5. In addition, YOLOv5 requires to set several hyperparameters that play a critical role to ensure optimal detection performance. Due to the complexity of hyperparamater setting, this operation is run through an appropriate optimization procedure whose details will be discussed below. Hyperparamater optimization as well as training and validation procedure require a training and validation set consisting of a sufficient number of images, where astrocyte locations have been annotated using rectangular bounding boxes in a specific format. In the workflow required by YOLOv5 to derive an optimized astrocyte detection model, training and validation data are first used to determine the optimal set of hyperparameters; after hyperparameters are set, training and validation data are again used to train and validate the network in order to derive the optimized astrocyte detection model. We remark that we have computed separate astrocyte detection models for the BBBC and Kruyer datasets. That is, we selected a training and validation set for each image dataset and we run separate procedures for hyperparameter optimization followed by network training and validation, resulting in two distinct astrocyte detection models. Clearly, each detection model is optimized for the image characteristics of the dataset on which it was trained.

#### Training and validation sets.

Images in each of the BBBC and Kruyer datasets were randomly split into a set for training, a set for validation and a set for testing. Specifically, for the BBBC dataset, 783 images were randomly assigned for training, 225 images for validation and 31 images for testing. For the Kruyer dataset, given the smaller size of the set, we changed the data split proportion and we assigned 169 images for training, 21 images for validation and 21 images for testing. In this case, since the images belong to different brain subregion and treatment, samples were drawn randomly and uniformly from each class.

For each training or validation image, we need to generate a label file storing the information about astrocyte locations. YOLOv5 requires the label file to store the coordinates of the rectangular boxes containing the objects of interest, i.e., the astrocytes, using PyTorch TXT format. In this format, each label is a vector of the form $(class, x\_center, y\_center, width, height)$, where *class* is the object class, $x\_center, y\_center$ are the *x* and *y* coordinates of the bounding box center and *width*, *height* are the width and height of the bounding box. These numbers are normalized by the dimensions of the image, that is

$$\begin{aligned} x\_center = \frac{box\_x\_center}{image\_width}, \quad y\_center = \frac{box\_y\_center}{image\_height}, \quad width = \frac{box\_width}{image\_width}, \quad height = \frac{box\_height}{image\_height}, \end{aligned}$$

where ${image\_height}$ and ${image\_width}$ are the height and width of the image, resp.; ${box\_height}$ and ${box\_width}$ are the height and width of the box, resp.; ${box\_x\_center}$ and ${box\_y\_center}$ are the *x* and *y* coordinates of the center of the box, resp.

For the Kruyer dataset, images were manually annotated by domain experts with labels stored in the requird PyTorch TXT format.

While the BBBC set includes image annotation, the label files are not consistent with the YOLOv5 PyTorch TXT format since label are in the form $(class, x_1, y_1, x_2, y_2)$, where $x_{i}$ and $y_{i}$ are x and y coordinates, resp., of the upper-left and lower-right corners of the bounding box in the image. To convert this file into the YOLOv5 PyTorch TXT format, we wrote a script in Python. For the Kruyer dataset, annotations originally saved in json format using the VIA software were converted into the YOLOv5 PyTorch TXT format using Roboflow^48^, a freely available conversion software.

#### Software and computational resources

All our numerical codes were implemented in Python 3.7 using the deep learning framework PyTorch 1.6.0. Our code, including the computed detection models is released in https://github.com/yewen/AstrocyteDetection; our annotated images are also included.

All computations needed to optimize the hyperparameters in YOLOv5 and then train the network to compute our astrocyte detection models were carried out using the Carya cluster from the University of Houston which houses public CPU and GPU nodes with shared access to storage resources, including a Tesla V100-SXM2-32GB GPU and a 48-core CPU.

#### Hyperparameter setting

YOLOv5 has about 25 hyperparameters that play a critical role in generating high-performance astrocyte detection. Such hyperparameters include learning rate, momentum, various parameters related to data augmentation and more. Since the application of image mosaic, image mixup and image scale for data augmentation may result in distortion of astrocyte shape and thus affect the correct learning process, we set to 0 the corresponding hyperparameters, meaning that the corresponding data augmentation transformations were not applied. Even after fixing these parameters, there are still many hyperparameters to be determined and finding optimal values for them is challenging. Traditional methods like grid searches are inefficient due to the high dimensional search space and the computational cost of evaluating the fitness at each point. For this reason, YOLOv5 includes a method of hyperparameter optimization, called Hyperparameter Evolution, that uses a Genetic Algorithm^49^ for hyperparameter optimization. Hyperparameter Evolution is resource intensive and time consuming since the network needs to be trained hundreds of times. In our setting, using the Carya computational cluster with 1 GPU and a 48-core CPU, it took about 20 hours to run Hyperparameter Evolution. Nevertheless, we found that the detection performance was significantly improved after applying Hyperparameter Evolution, as compared with default hyperparameter setting, with Dice Coefficient performance score (defined below) increasing about 15%.

#### Computation of detection models

After running Hyperparameter Evolution and computing the best hyperparameters for our astrocyte detection network, we still had the option to modify the weights of the Fitness Function before training our network. We ran extensive numerical experiments with different weights assignments and found the best performance to occur with weights of (0.25, 0.25, 0.05, 0.45) for the BBBC dataset and weights of (0.20, 0.20, 0.30, 0.30) for the Kruyer dataset.

**Figure 2:**
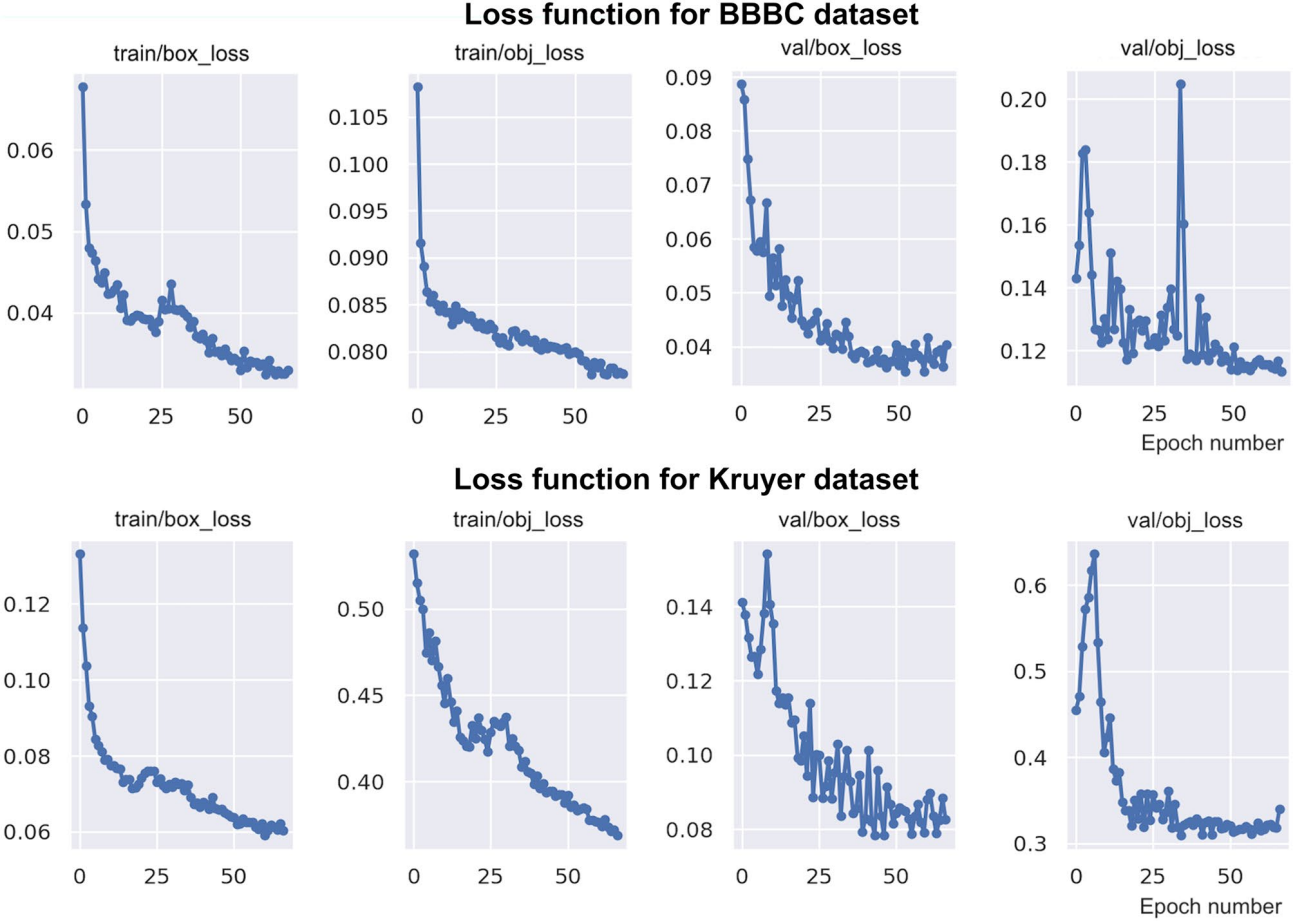
Loss function. Plots display location or bounding box losses (box_loss) and objectness losses (obj_loss) as a function of the number of epochs during training and validation for the BBBC (top) and Kruyer (bottom) datasets.

Figure 2 shows the location and the objectness loss functions evaluated during training and validation after hyperparameter optimization. The plots show that all loss function curves decrease as the number of epochs increase and reach a minimum after about 65 epochs. YOLOv5 automatically stops the training process if there is no additional reduction of the loss function. We found that, for the BBBC dataset, the box and objectness losses converge to 0.0330 and 0.0776 respectively; for the Kruyer dataset, the box and objectness losses converge to 0.0603 and 0.3687 respectively. We attribute the larger value of the loss function for the Kruyer dataset compared to the BBBC dataset with the greater difficulty in computing an accurate detection model, given the more heterogeneous nature of astrocyte images in the Kruyer dataset that includes cells from different brain regions and under different physiological conditions.

For both datasets, during training and validation, we used the Adam optimizer in the standard configuration. For the BBBC dataset, we chose batch size 32, while for the Kruyer dataset—where images are larger—we chose batch size 16. We found heuristically that these batch sizes provided the best performance. Each detection model was run for up to 300 Epochs. However, YOLOv5 includes an early stop mechanism that interrupts the training process if the model stops improving after a given epoch; as a result, the training was stopped at 66 epochs for the BBBC dataset and at 67 epochs for the Kruyer dataset.

### Evaluation metrics

To assess detection and segmentation performance, we used the standard binary classification metrics of sensitivity, precision and Dice coefficient^50^.

The *recall* (or True Positive Rate or *sensitivity*) measures the proportion of correctly detected cells (or points) with respect to the total number of cells that were manually identified by a domain-expert without knowledge of the algorithm results. Denoted by TP (= true positive) the number of correct detections and by FN (= false negative) the number of missed detections, we define the sensitivity as

$$\begin{aligned} \text {R = } \tfrac{TP}{TP + FN} \end{aligned}$$

The *precision* measures the proportion of correctly detected cells over all detected cells. That is, denoting by FP (= false positive) the number of wrongly detected cells, the precision is

$$\begin{aligned} \text {P = } \tfrac{TP}{TP + FP} \end{aligned}$$

Finally, the *Dice Coefficient* (also known as *F1 score*) can be considered a measure of the overall effectiveness of the classification algorithm and is given by

$$\begin{aligned} \text {DC = } \tfrac{2TP}{2TP + FP + FN}. \end{aligned}$$

DC ranges between 0 and 1 with $DC=1$ describing perfect classification.

Precision–Recall (PR) curves illustrate how a model’s precision (y-axis) varies as a function of recall (x-axis) and are extensively used for evaluating classifiers in machine learning, especially in the situation of a single-class classifier^51^. A higher curve in the PR space corresponds to a superior classification performance of the algorithm.

Each point in PR space represents a specific classifier associated with a threshold value for calling an example positive. In our experiments, this threshold is the confidence threshold that we introduced above in relationship with the detection score computed by the YOLO-based algorithm. Clearly, any threshold value determines a trade-off between false positives and false negatives, with higher threshold values associated with less false positives (hence higher precision) and, viceversa, lower threshold values associated with less false negatives (hence higher recall).

## Results

In this section, we illustrate the application of our method based on YOLOv5 for the automated detection of astrocytes.

As indicated above, we tested our algorithm using 2 image datasets posing different types of challenges. Images in the BBBC dataset were more homogeneous than the Kruyer dataset, as the Kruyer image set included cells acquired from different subregions of the brain and cells exposed to perturbations possibly affecting cell morphology and cell density. In fact, a simple calculation shows that, in the BBBC dataset, there were about 10-30 cells per image; by contrast, there were between 6 to 65 cells per image in the Kruyer dataset. Image contrast was also different since the BBBC dataset includes brightfield microscopy images while the Kruyer dataset includes images acquired from high-resolution fluorescent microscopy.

To assess the performance of our method for astrocyte detection, we tested 31 images from the BBBC dataset containing a total of 484 cells and 21 images from the Kruyer dataset containing a total of 565 cells. The test images were randomly selected from their respective datasets. For the Kruyer datset,—which includes images of cells from different brain subregions exposed to different perturbations—we selected the test images uniformly from each brain subregion and perturbation class.

To benchmark our YOLO-based algorithm performance against existing and state-of-the-art algorithms, we considered the following astrocyte detection methods that were applied to the same test images.

1. Adaptive Thresholding^25^. This method was implemented using the functionalities of the widely-used Fiji software^26^ and includes the following steps. Images were first pre-processed using rolling ball background subtraction and the despeckle function for noise removal. Next, images were processed with intermodes threshold generating a binary image that separated the background from the set of potential cells contained in the image. Since the output of the thresholding step may include objects that are too small to be complete cells (e.g., isolated cell processes), this output was further processed by removing objects that were significantly smaller than the average cell size using the MATLAB command bwareaopen. Specifically, we removed objects that were smaller than 180 pixels for the BBBC dataset and 155 pixels for the Kruyer dataset, where the values were determined by assessing the average astrocyte size in each dataset.
2. GESU-net^31^. This approach is an algorithmic pipeline for astrocyte detection and segmentation. Input images were denoised using a multiscale approach before the cell detection step, which was implemented using a multiscale geometric descriptor, called Directional Ratio. This method detects potential cells by automatically separating cell bodies, even for cells having irregular shapes, from the rest of the image. The algorithm includes a postprocessing routine that discards objects not satisfying prescribed cell properties (e.g., size, shape).
3. FindMyCell^30^. This deep learning framework based on DetectNet was developed specifically for astrocyte detection and was trained using images from the BBBC dataset. Unfortunately, its code is outdated and we were unable to run the program in the present language environment. However, we have access to astrocyte detection results of the software for a few selected images from the BBBC dataset that we could use to run a comparison.

We remark that, for all our methods, we ignored cells that were overlapping the boundary of the image, since we expected that the truncation of the cell due to the image boundary may affect cell morphology causing inconsistencies with cells that are fully contained inside the image.

**Figure 3:**
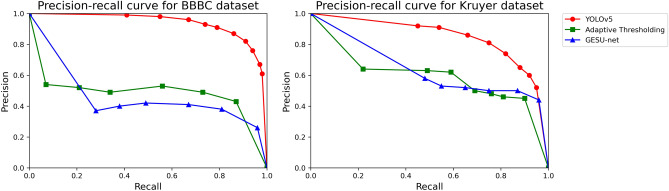
Precision–recall curves. Precision–recall curves demonstrate the astrocyte detection performance of different algorithms on the BBBC (left panel) and Kruyer (right panel) image datasets. Our method based on YOLOv5 is compared against GESU-Net^31^ and Adaptive Thresholding implemented using FIJI (see description in the text).

Figure 3 compares the astrocyte detection performance of our method against Adaptive Thresholding and GESU-net on the BBBC and Kruyer datasets using Precision–Recall curves. We remark that this performance metric provides a rigorous assessment of the overall performance of a detection algorithm by illustrating the trade-off between the true positive rate (recall) and the positive predictive value (precision) for a predictive model using different probability thresholds. A high area under the curve indicates both low false positive and low false negative rates so that the higher is the curve, the better is the detection performance. Figure 3 shows that our YOLO-based approach exhibited an excellent performance overall, significantly outperforming Adaptive Thresholding and GESU-net for any recall value. Our method appears to perform slightly better on the BBBC dataset than the Kruyer dataset where the curve was lower. We explain this slight difference in performance as a consequence of the increased heterogeneity within the Kruyer dataset which includes astrocytes under different conditions and from different brain locations. Adaptive Thresholding and GESU-net perform rather poorly, especially on the BBBC dataset where image contrast was lower.

Table 1 compares the astrocyte detection average performance of our method against Adaptive Thresholding and GESU-net on the BBBC and Kruyer datasets. We chose to compare average recall and precision rates corresponding to the best Dice coefficient for every method. Our method achieved higher values of Precision and Recall rates resulting in high value of the Dice coefficient. As observed and explained above, our method performed better on the BBBC dataset than the Kruyer dataset. Both Adaptive Thresholding and GESU-net show low precision, especially on the BBBC dataset indicating a high number of false positive detections. The best Dice coefficients for both methods were significantly lower than the best Dice coefficient for YOLOv5. These comparisons are consistent with the Precision–Recall curves in Fig. 3 that are dominated by the PR-curve for our customized YOLOv5 detection algorithm.

**Table 1:** Astrocyte detection performance corresponding to best Dice coefficient of our algorithm based on YOLOv5, Adaptive Thresholding and GESU-net^31^.

Dataset	YOLOv5			Adaptive thresholding			GESU-net		
	R	P	DC	R	P	DC	R	P	DC
BBBC (484 cells)	0.87	0.86	0.86	0.73	0.49	0.58	0.84	0.38	0.52
Kruyer (565 cells)	0.75	0.81	0.78	0.59	0.62	0.60	0.87	0.50	0.63

Performance metrics include Recall (R), Precision (P) and Dice Coefficient (DC).

As explained above, we could not carry out a complete comparison with FindMyCell but we have access to the astrocyte detection results of the software for 11 images from the BBBC dataset, including a total of 188 cells. On those images, FindMyCell achieves Recall = 0.70, Precision = 0.86, Dice coefficient = 0.77 and this result is comparable with the published results^30^. On the same images, our method based on YOLOv5 achieves Recall = 0.85, Precision = 0.78, Dice coefficient = 0.82, showing that our method outperforms FindMyCell with respect to Recall and Dice coefficient. Since the Dice Coefficient is a measure of overall performance and our Dice coefficient value is about 6% higher, this suggests that our method performs slightly better than FindMyCell on the BBBC dataset. It is unfortunate that we could not compare performances on the more challenging Kruyer dataset.

**Figure 4:**
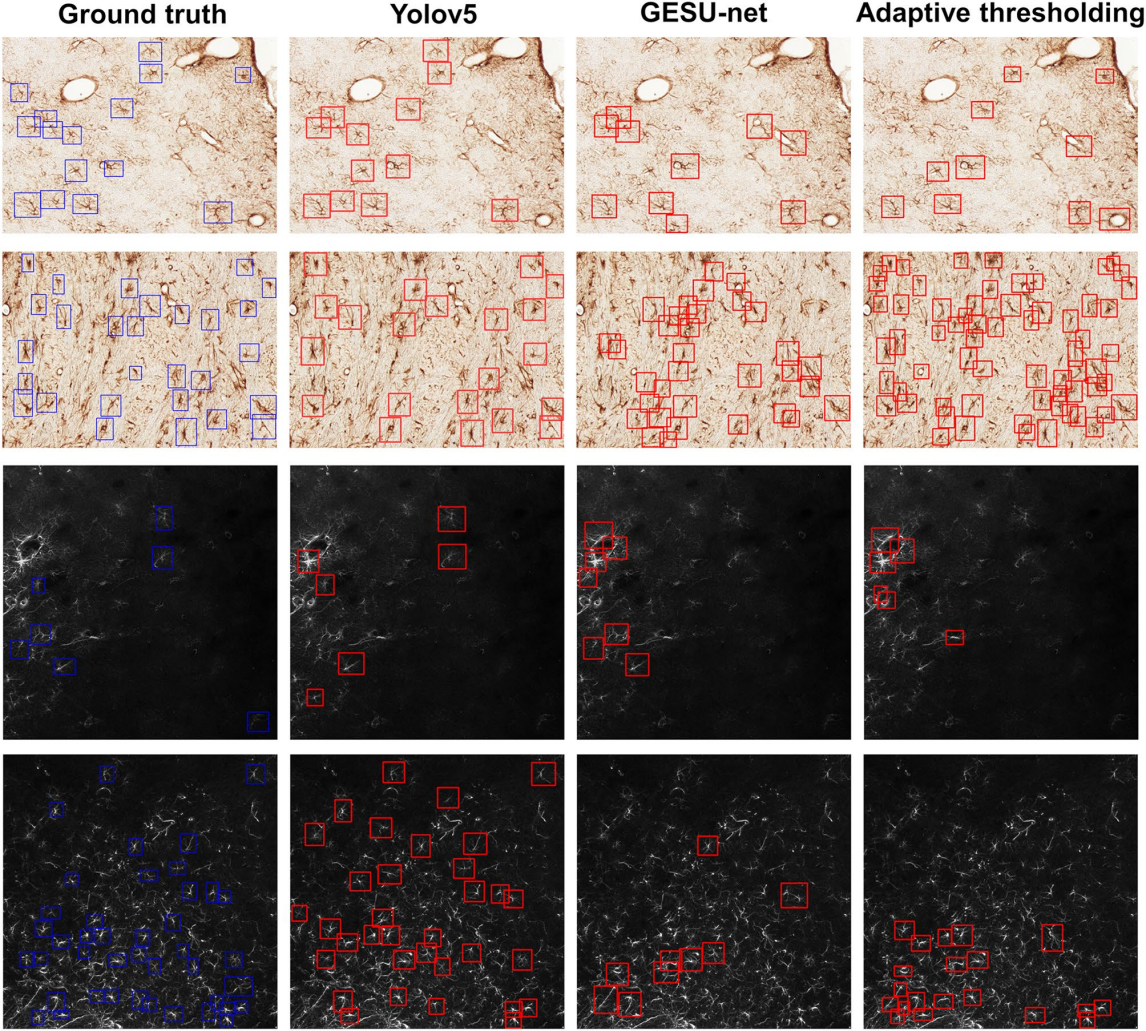
Detection performance. Astrocyte detection performance using different algorithm is shown on two representative images from the BBBC dataset (top two rows) and two representative images from the Kruyer dataset (bottom two rows). The first column shows the ground truth images, the second column shows the corresponding detection results of using our algorithm based on YOLOv5 (with probability threshold 0.47), the third column shows the detection result of the GESU-net algorithm and the fourth column shows the detection result using Adaptive thresholding implemented in FIJI.

To further illustrate the properties of our detection algorithm, Fig. 4 compares the astrocyte detection performance of our method against Adaptive Thresholding and GESU-net on 4 representative images from the BBBC and Kruyer datasets with different cell densities. The figure shows that our YOLO-based approach performed consistently well on both datasets for images with both low and high population densities. While both Adaptive Thresholding and GESU-net performed significantly worse than our approach, the difference in performance was more striking for high-density images. For Adaptive Thresholding, the difficulty in handling images with higher population density is explained by the expected difficulty in setting a threshold that separates astrocytes from the background without also including objects that are not cells. This difficulty is exemplified by the large number of false positive detections in the second row, fourth column, and by the large number of false negative detections in the fourth row, fourth column in Fig. 4. GESU-net on the other hand is designed to detect astrocytes that have recognizable cell bodies, so that it missed a large number of cells (false negative detections).

### Algorithm run time

We compared the algorithm run time using a MacBook Pro with a 2.3 GHz Quad-Core Intel Core i5 and 8 GB 2133 MHz LPDDR3 memory. Our algorithm based on YOLOv5 for astrocyte detection took approximately 0.03 seconds to process a single image. By contrast, the time required to process a single image was about 0.64 seconds for Adaptive Thresholding and about 0.50-1.00 seconds for GESU-net, where the computing time depends on the number of cells present in the image.

### Statistical analysis

**Figure 5:**
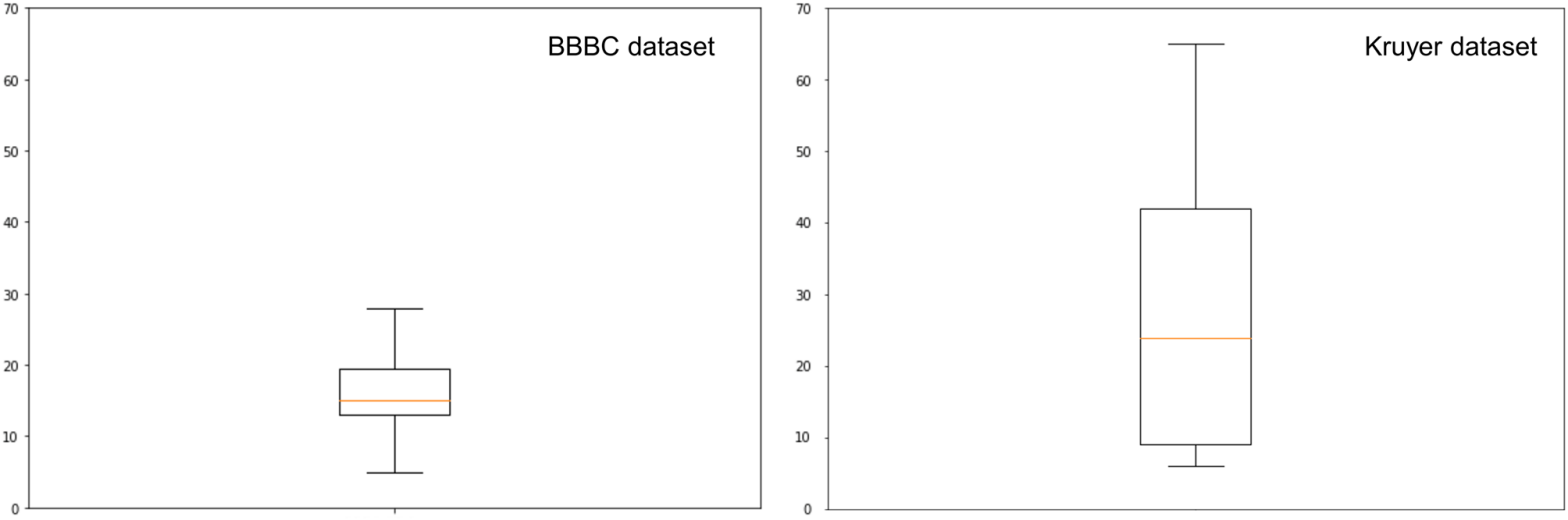
Cell count distribution. The boxplots show the different cell count distributions in the BBBC and Kruyer datasets. As usual, the box shows the interquantile range with the line inside indicating the median value.

We carried out a more careful statistical analysis of our algorithm to better assess how it performed on different images. Our first observation was that the cell count in the images varied resulting in a positively skewed empirical distribution with an inter-quantile range (IQR) which was wider for the Kruyer images as compared to the BBBC images, as shown in Fig. 5. Nonetheless, we observed that both image datasets could be partitioned into three classes depending on cell densities: images containing relatively few astrocytes, that we called *sparse*; images containing a dense population of cells, that we called *dense*; images whose cell density was in-between, that we called *intermediate*. Specifically, we designated as sparse the images with astrocyte counts not exceeding the 1st quartile of the cell count distribution (Q1), as intermediate those images with counts in the interquartile range (IQR), and as dense images with counts in the top quartile.

We next investigated the possible variation of recall and precision rates for a given detection threshold among different image subclasses. To carry out this analysis, we computed the confidence interval estimation for the recall and precision rates using two methods: *exact binomial test*^52^ and *bootstrapping*^53^. When the methods were applied, we considered any cell detection event to be independent from any other cell detection event, even if both cells were in the same image. We also remark that non-parametric bootstrapping applies to the situation where the sample sizes in the test sets are not equal, corresponding to our setting. Let us briefly explain how we performed the bootstrap estimation for the confidence interval for any image subclass in a test set. Suppose that this subclass has a total number of *k* ground truth cells, counting all images. Given a threshold, suppose that we detected $l \le k$ of those cells. We generate a large number of samples (typical size is 10,00) of size *k* by repeatedly sampling with replacement from a “bag” containing *l* 1’s and $k-l$ zeroes, where 1 indicates that a cell is detected and 0 that is not. Our statistic is defined as the relative frequency of detections in each of the *k*-sized samples. The non-parametric bootstrap confidence interval is then calculated from the empirical distribution defined by these 10,000 samples, which we use as the sampling distribution of the recall or precision parameters. The 95% bootstrap interval is defined by the 2.5% and 97.5% percentiles of this empirical distribution.

Table 2 reports the 95% confidence intervals of the precision and recall rates by our algorithm on the BBBC and Kruyer datasets computed using exact binomial test and bootstrapping. These values were computed using the precision and recall point estimates for the best Dice coefficient that were used for Table 1. Results in the table show that bootstrapping yielded a more narrow confidence interval and that recall and precision rates varied between image subclassses of each dataset. Comparison of the confidence intervals confirms that our algorithm performs better on the BBBC than the Kruyer dataset. The bootstrap approach shows that our algorithm exhibited a slightly higher recall rate on dense images for the BBBC dataset and higher recall rate on sparse images on the Kruyer dataset; vice versa, it exhibited a slightly higher precision rate on sparse images for the BBBC dataset and higher precision rate on dense images on the Kruyer dataset. Overall our algorithm performed very consistently on the BBBC dataset for all image types. On the other hand, for the Kruyer dataset, our algorithm performed slightly better on dense images rather than sparse ones. We explain this performance with the observation that sparser images in the Kruyer dataset typically corresponded to astrocytes from animal models of heroin withdrawal and relapse, where cell morphology may be affected (this observation is consistent with findings from the literature^22^), leading to a lower precision rate.

**Table 2:** Confidence intervals of recall and precision rates on the BBBC and Kruyer datasets using our YOLO-based algorithm.

Image density	Recall		Precision	
	BBBC dataset	Kruyer dataset	BBBC dataset	Kruyer dataset
Sparse	[0.727, 0.885]	[0.686, 0.922]	[0.888, 0.988]	[0.519, 0.775]
	[0.795, 0.839]	[0.814, 0.860]	[0.939, 0.966]	[0.601, 0.659]
Intermediate	[0.841, 0.932]	[0.710,0.850]	[0.857, 0.944]	[0.734, 0.867]
	[0.871, 0.906]	[0.801, 0.848]	[0.863, 0.907]	[0.793, 0.844]
Dense	[0.832, 0.928]	[0.682, 0.774]	[0.854, 0.944]	[0.789, 0.873]
	[0.870, 0.906]	[0.715, 0.776]	[0.890, 0.931]	[0.818, 0.865]

Each table cell reports the confidence intervals calculated using exact binomial method (top) and bootstrapping (bottom).

## Discussion

Automated detection of astrocytes in 2D microscopy images is a very challenging problem for which very few methods are currently available in the literature.

In this paper, we have introduced a new approach that exploits the expressive power of YOLOv5—an advanced deep learning framework for object detection—to provide state-of-the-art astrocyte detection performance in brightfield and fluorescent micrographs. YOLOv5 offers a highly flexible platform that can manage multiple images sizes, is highly customizable, is implemented in PyTorch, the most widely used software library in the research community, and can be trained with a relatively small number of labeled data.

To benchmark our detection approach, we have compared it against Adaptive Thresholding implemented using FIJI, GESU-net—a method that uses multiscale filters—, and FindMyCell (FMC)—a deep-learning method specifically designed for astrocyte detection. Unfortunately, it was not possible to carry out a complete comparison with FindMyCell as the software is outdated and cannot be run at this time; we could only provide a limited comparison using a small number of test images on which the performance of FindMyCell had been recorded. The algorithm performance was quantitatively assessed using Precision–Recall curves, as well as the Dice Coefficient. Our numerical experiments have shown that our YOLO-based approach performed significantly better than Adaptive Thresholding and GESU-net, especially for images with a dense cell population, where both thresholding and filter-based methods are unable to accurately separate astrocytes from the background. Since supervised learning strategies are generally expected to perform better than traditional model-based methods in image classification tasks, this performance improvement was expected. Our method was also shown to be very competitive against FindMyCell, another deep learning method. Even though we could only run a comparison on a small set of images from the BBBC dataset, on this set we found that our approach exhibits a slightly improved detection performance. We did not carry out any additional comparison against general-purpose algorithms for cell detection, e.g., ILASTIK^54^, as it was shown by some of the authors that such methods do not perform better than Adaptive Thresholding when applied to images of astrocytes^31^.

To better assess the reliability of our algorithmic approach, it was tested using two datasets with different image contrasts and cell populations. A detailed statistical analysis shows that the performance of our algorithm is remarkably robust, yielding a low number of misclassifications. On the BBBC dataset, its performance was stable overall across images with different population densities. On the more challenging Kruyer dataset, where the images are less homogeneous as they include astrocytes from animals under behavioral perturbation which may affect astrocyte morphology, the performance was remarkably consistent. In fact, for the Kruyer dataset, we observed slightly better performance in images with high population densities as compared with images with relatively low density. We explain this difference in performance with the fact that low-density images are more likely to be from animals under behavioral perturbation, with the consequence that the possibly different morphological characteristics of these astrocytes is less represented in the training set, as compared to the astrocytes from images with higher population density.

**Figure 6:**
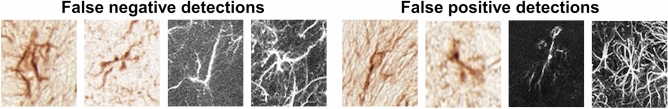
Examples of misclassification. Representative sub-images extracted from the BBBC and Kruyer datasets show examples of astrocytes that where missed (false negative detections, first four columns) or incorrectly identified (false positive detections, last four columns).

Figure 6 shows some representative examples of misclassified cells resulting from our algorithm on the test images we considered. A close inspection of these images suggests that: (i) false negative detection typically occurs for objects that are not well represented in the training set either because their morphology is rare or because it is similar to objects often assigned to the background; (ii) false positive detection most often occurs for objects that appear similar to astrocytes and could have been missed in the ground truth by the image annotators. These observations underlines the challenge to generate accurately-annotated images of astrocytes. In fact, we observed different domain experts to assign conflicting labels for an object more frequently than in similar tasks concerning other types of cells. This difficulty explains perhaps the lack of publicly annotated images of astrocytes which is a main factor hampering the development of accurate algorithms targeted to their automated detection. For this reason, we are convinced that the public release of our Kruyer dataset will be highly beneficial to the scientific community.

We remark that our strategy to design a YOLO-based algorithm for the automated detection of astrocytes can be easily adapted to other types of glial cells, where similar challenges are typically found. To do so, one would have to compute a new cell detection model which would require to generate a suitable annotated image dataset for training and validation. Since YOLO can handle multi-class detection tasks, it can also be trained to detect different classes of cells (assuming they exhibit some distinct morphology) and assign them to the respective class. For instance, as the GFAP marker is not fully specific, e.g., during development both astrocytes and radial glia express GFAP, one could develop a similar YOLO-based algorithm to detect the two types of cells as two distinct types of objects. Again, to do so would require to generate a suitable annotated image dataset to carry our training and validation.

To conclude, our algorithm for astrocyte detection provides a fundamental tool for the quantitative analysis of astrocytes. As shown by some of the authors^31^, astrocyte detection can be integrated into existing routines of cell segmentation to extract morphological characteristics of astrocytes at the single-cell level, providing a critical tool for the development of automated algorithms for astrocyte profiling or classification.

## Data Availability

The Kruyer dataset is available at https://github.com/yewen/AstrocyteDetection. The BBBC datas et is available at https://data.broadinstitute.org/bbbc/image_sets.html. Our python scripts and classification models are also available at https://github.com/yewen/AstrocyteDetection.
